# Parents' Beliefs and Behaviors About Their Children's Literacy Development: A Cross-Sectional Study in Saudi Arabia

**DOI:** 10.3389/fped.2022.708217

**Published:** 2022-04-07

**Authors:** Fatimah Saeed AlAhmari

**Affiliations:** Department of Pediatrics, King Faisal Specialist Hospital and Research Center, Riyadh, Saudi Arabia

**Keywords:** literacy beliefs, Saudi Arabia, literacy behaviors, children literacy, Perceptions of Literacy Learning Interview Schedule

## Abstract

**Background:**

A home environment that promotes learning is a significant factor in school performance in which children's parents are involved in their education. However, little study has focused on parents' reading-related beliefs or the relationship between parents' literacy beliefs and behaviors.

**Methods:**

This cross-sectional study describes the range of parents' reported literacy beliefs and behaviors and examines whether an association exists in Saudi Arabia. It was conducted on a convenient sample of 100 parents using a web-based self-administrated shared with families in Developmental and Behavioral Pediatrics clinics at multiple sites utilizing the Parents' Perceptions of Literacy Learning Interview Schedule (PPLLIS).

**Results:**

There were 83 mothers and 17 fathers; 90% had a university degree or higher. Parents' beliefs ranged between 87 and 123, with a mean of 103.54 ± 8.05, indicating more holistic perceptions than skills-based ones. There was only a positive significant correlation between parents' beliefs and behaviors regarding literacy activities. However, those participating in literacy activities with their kids were more holistic parents and scored the top one-third on the PPLLIS.

**Conclusion:**

Parents' beliefs and their reported behaviors are found to be significantly impacted by their educational levels. Therefore, future investigations and national campaigns are encouraged to improve parents' educational levels, especially in urban areas.

## Introduction

Literacy is defined as having the ability to read and write as introduced within a significant context (1). Many techniques to improve literacy skills and enhance the significance of outcomes among children have been previously reported. For instance, exposure to storybooks and reading shared books are some of the commonly studied literacy aspects. However, evidence regarding the efficacy of these modalities in enhancing literacy skills is controversial among studies in the literature (2, 3). Among children, it has been previously reported that early acquisition of literacy skills within the first 5 years of life can significantly enhance the academic performance of these children (4, 5).

On the other hand, it has also been reported that lacking these skills can negatively impact the child's academic performance and significantly persist through adolescence (6, 7). However, parents' involvement in literacy development in their children is a fruitful practice with favorable outcomes on education (8). Leslie et al. (9) previously showed that parents' involvement in children's literacy activities was associated with a significant enhancement in the educational outcomes in elementary school children. It has been reported that less involvement with preschool children and reduced supervision periods from the caregivers was associated with the reduced acquisition of essential phenomics and vocabulary skills (10). Parents' beliefs may fluctuate on how children can become literate regarding their study and literacy education knowledge. Previous evidence revealed that when parents were questioned about their beliefs about learning, reading, and writing, it mainly occurred when children were already in regular education (11, 12).

The role of the family has become of great importance in educating preschool children, almost equivalent to the educational services offered at schools. However, little study has focused on parents' reading-related beliefs or the relationship between parents' literacy beliefs and behaviors. Understanding parents' beliefs about literacy is vital in identifying the children's home environment and activities parents engage in. In Saudi Arabia, reports show that many families have recently begun to be interested in enrolling their children in kindergarten at least 1 year earlier, despite being non-mandatory at that age group (13). However, no previous studies have been conducted in Saudi Arabia to assess parents' beliefs and attitudes toward literacy enhancement practices. Therefore, this study describes the range of parents' reported literacy beliefs and behaviors and examines whether an association exists in Saudi Arabia.

## Methods

### Study Design and Data Collection

This cross-sectional study was conducted on a convenient sample of 100 parents using a web-based self-administrated shared with families at Developmental and Behavioral Pediatrics clinics in various private or governmental institutes around Saudi Arabia. Initially, the sample size was obtained through cluster sampling methods with an expected response rate of 90% and a sample size of 145. However, only 100 parents were involved in the final study after applying the inclusion and exclusion criteria. Inclusion criteria were all the children attending the behavioral pediatric clinics between March and June 2021 and who are regularly following up while those who are not known to regularly follow-up in the clinic or have an incomplete file were excluded. Data were collected by medical student volunteers from secondary and tertiary hospitals between March and June of 2021. All parents consenting to undertake the survey were included, and couples with no children were excluded. The survey utilized the Parents' Perceptions of Literacy Learning Interview Schedule (PPLLIS) (14, 15) questionnaire, and answers were collected in an excel sheet. This tool evaluated parents' beliefs on how children learn to read and write and gave information on the varieties of literacy exercises parents are involved in with their kids. Given the responses, parents' perceptions of literacy were described as more emergent or more traditional. We modified the PPLLIS score by excluding three questions (items 3, 4, and 27) (Supplementary Material 1). All the questions were scored on a 5-point Likert scale (strongly agree to strongly disagree), with a greater score given for answers that represented a more emergent or holistic literacy perspective (strongly agree scored 1 and strongly disagree 5 except for questions: 1, 2, 3, 6, 7, 8, 16, 17, 19, 20, 22, 23, 24, 25, 26, and 28 where strongly agree scored 5 and strongly disagree scored 1). Scores were calculated by summing the raw scores, which means the lowest possible score on the questionnaire is 30 and the highest possible score is 150.

One question included in the questionnaire to assess “parents' beliefs” was asking the parents to name the five most important ways they help their children learn to read and write. The responses were scored based on the frequency and were categorized into five categories based on the answers. The first category: teaching literacy skills, included parents' direct attempts to instruct children about reading and writing. Second category: being involved in supporting children's literacy by encouraging and valuing children's literacy development. Third category: participating in literacy activities included literacy events in which parents and children collaboratively participated. Fourth category: knowledge development had parents' attempts to promote children's general intellectual or cognitive development. Fifth category: involving those who did not fit any of the previous categories.

### Statistical Analysis

Reports were collected, then coded and revised, and data were introduced on statistical software IBM SPSS version 26.0. All the statistical analysis was done using two-tailed tests and an alpha error of 0.05. A *P*-value < 0.05 was considered to be statistically significant. Frequencies and percentages were used to describe the distribution of items and scale. Finally, the questions were assessed and scored. The Cronbach's alpha test was used for overall ranking and objects and the Cronbach's alpha coefficient of more than 0.70 was considered acceptable. In this study, the Cronbach's alpha was 0.682, indicating an average internal consistency for our scale with this specific sample.

To facilitate data analysis, the included parents were grouped based on their PPLLIS score. Out of the 100 parents that completed the questionnaire, 32 parents scored the lowest on the PPLLIS and were placed in group 1. The following 33 parents whose scores fell in the middle were placed in group 2. Meanwhile, the 35 parents whose holistic beliefs were high and scored in the top third on the PPLLIS were placed in group 3. Finally, the groups were as follows; group 1: more skills-based group with the lowest scores, group 2: combination group of both the skills-oriented and holistic beliefs, in comparison to the other groups, group 3: more holistic orientation to literacy learning with the highest score.

The Shapiro–Wilk test was used to verify the normal distribution of continuous variables. For normally distributed variables, the ANOVA test was used to examine whether parents' education was examined differed in parents' beliefs. The Pearson's chi-squared was used to assess the differences between categorical variables. The Fisher's exact test was used in the cases where the conditions were not satisfied (≥20% of the expected values are <5 and the minimum is <1). In addition, partial correlations were used to examine relationships between parents' beliefs and their behaviors, controlling for children's age (15). The control for the age was done because parents' behaviors may vary with children's age (16).

## Results

### Participant's Characteristics

Overall, 100 participants completed the data. Table 1 shows participants' sociodemographic characteristics. There were 83 mothers and 17 fathers involved in this study and most of them were from the eastern province of Saudi Arabia (78%). Their education level ranged from high school completion to doctorate degrees, with 90% having a university degree and higher education. On average, the children in this study had the older sibling's mean age of 12.33 ± 7.75 years, while the younger sibling was 4.49 ± 3.82 years. In addition, 66 participants spoke Arabic in the house, while 34% of parents spoke another language.

**Table 1:** Socio-demographic and educational characteristics of the participants.

**Variable**	**Count**	**%**
**Parent's sex**
Male	17	17
Female	83	83
**Province**
Aseer	2	2
Eastern	78	78
Qassim	4	4
Makkah	7	7
Madinah	5	5
Riyadh	4	4
**Highest education of the parent**
None	1	1
High school	9	9
University and higher	90	90
Older sibling age (Mean ± SD)	12.33 ± 7.75
**Older sibling sex**
Male	54	54
Female	46	46
Younger sibling age (Mean ± SD)	4.49 ± 3.82
**Younger sibling sex**
Male	54	54
Female	46	46
**Languages spoken at home**
Arabic	66	66
Arabic + other	34	34
**Languages spoken by the child**
Arabic	60	60
Arabic + other	40	40

### Parent's Beliefs

Table 2 describes the overall participants' scores. Parents' beliefs ranged between 87 and 123, with a mean of 103.54 ± 8.05, indicating more holistic perceptions than skills-based ones (a higher score). The following five statements on the PPLLIS were those on which parents scored the lowest: real reading begins only when a child starts to say the words as they are printed on the page, a child can begin to write before she has learned the correct spelling of the terms, a child can begin to write (e.g., notes and stories) before she knows how to read, only gifted children learn to read and write before receiving formal instruction in preschool or elementary school, schools should be responsible for teaching children to learn to read and write, and children have to be of a certain age before they can begin to learn to read and write.

**Table 2:** The Parents' Beliefs of Literacy Learning Interview Schedule (PPLLIS) score.

**Number**	**Questions**	**Strongly agree**	**Agree**	**Neutral**	**Disagree**	**Strongly disagree**
1	A child learns to read by first learning the letters of the alphabet and their sounds, then words, then sentences and then stories.	44 (44%)	37 (37%)	14 (14%)	4 (4%)	1 (1%)
2	Teaching a child to recognize isolated words on sight is a suitable technique for teaching her to read.	21 (21%)	53 (53%)	15 (15%)	9 (9%)	2 (2%)
3	A child benefits from hearing favorite stories that she has memorized read again and again.	58 (58%)	40 (40%)	2 (2%)	0 (0%)	0 (0%)
4	You should not encourage a child to join in sometimes while you read a book with which he is familiar for is it better that the child listens to the story without interruption.	7 (7%)	8 (8%)	20 (20%)	44 (44%)	21 (21%)
5	You will be teaching your child a bad habit if you point to the print as you read.	3 (3%)	9 (9%)	21 (21%)	43 (43%)	24 (24%)
6	You are helping a child learn to read by encouraging her to discuss what is being read.	60 (60%)	35 (35%)	4 (4%)	1 (1%)	0 (0%)
7	It is necessary to check a child understands by asking him questions at the end of each story.	47 (47%)	41 (41%)	9 (9%)	3 (3%)	0 (0%)
8	You should permit your child to read familiar books by retelling the story from memory using the pictures.	36 (36%)	54 (54%)	10 (10%)	0 (0%)	0 (0%)
9	Real reading begins only when a child begins to say the words as they are printed on the page.	6 (6%)	32 (32%)	27 (27%)	23 (23%)	12 (12%)
10	It is necessary for a child to know the letters of the alphabet, and the sounds of the letters of the alphabet before she begins to write.	47 (47%)	26 (26%)	21 (21%)	3 (3%)	3 (3%)
11	A child should learn to print neatly the letters of the alphabet before attempting to print messages, notes, stories and so forth.	42 (42%)	29 (29%)	16 (16%)	12 (12%)	1 (1%)
12	It is necessary for a child to have lots of experience copying words, then sentences, and finally stories before she attempts to write on her own.	24 (24%)	36 (36%)	23 (23%)	13 (13%)	4 (4%)
13	A child should be encouraged to write only easy words and short sentences when he begins to write.	41 (41%)	45 (45%)	10 (10%)	2 (2%)	2 (2%)
14	A child's early scribbling are related to later development in writing stories, messages, etc.?	37 (37%)	48 (48%)	12 (12%)	3 (3%)	0 (0%)
15	A child needs workbooks to learn how to write.	39 (39%)	42 (42%)	14 (14%)	4 (4%)	1 (1%)
16	A child can begin to write before she has learned the correct spelling of the words.	19 (19%)	33 (33%)	24 (24%)	23 (23%)	1 (1%)
17	You SHOULD correct your child if she writes “kt” for the word “cat.”	38 (38%)	46 (46%)	11 (11%)	5 (5%)	0 (0%)
18	A child's confusion of “b” and “d” or “p” and “q” in printing indicates a major problem	2 (2%)	11 (11%)	32 (32%)	33 (33%)	22 (22%)
19	A child can begin to write (e.g., notes, stories) before she knows how to read.	5 (5%)	22 (22%)	25 (25%)	38 (38%)	10 (10%)
20	Learning to read and learning to write are similar to learning to talk in that children learn these skills gradually.	37 (37%)	59 (59%)	2 (2%)	2 (2%)	0 (0%)
21	Only gifted children learn to read and write before receiving formal instruction in preschool or elementary school.	13 (13%)	26 (26%)	33 (33%)	20 (20%)	8 (8%)
22	Reading to, and with children helps them learn to write	43 (43%)	48 (48%)	9 (9%)	0 (0%)	0 (0%)
23	Children learn important things about reading and writing before they begin formal reading programs at preschool or elementary school.	31 (31%)	44 (44%)	21 (21%)	4 (4%)	0 (0%)
**These activities help children learn to read and to write:**						
24	Talking to them.	56 (56%)	39 (39%)	4 (4%)	1 (1%)	0 (0%)
25	Having them pretend to write grocery lists with you.	56 (56%)	36 (36%)	7 (7%)	1 (1%)	0 (0%)
26	Reading to them	56 (56%)	36 (36%)	6 (6%)	2 (2%)	0 (0%)
27	Schools should be totally responsible for teaching children to learn to read and to write.	10 (10%)	8 (8%)	24 (24%)	41 (41%)	17 (17%)
28	It is very important that children see their parents reading and writing.	54 (54%)	38 (38%)	7 (7%)	1 (1%)	0 (0%)
29	Children have be certain age before they can begin to learn to read and write.	10 (10%)	23 (23%)	34 (34%)	26 (26%)	7 (7%)
30	Children need training in hand-eye coordination recognizing shapes, and so forth before they begin to learn recognizing shapes, to read and to write.	35 (35%)	55 (55%)	9 (9%)	1 (1%)	0 (0%)
**Total Score (Mean** **±SD)**		103.54 ± 8.05				

Parents scored highest on the following five statements of the PPLLIS: a child benefits from hearing favorite stories that she has memorized read, again and again, you are helping a child learn to read by encouraging her to discuss what is being read, talking to them, having them pretend to write grocery lists with you, and reading to them. However, when testing the associations between the PPLLIS score and different sociodemographic characteristics, none of the variables was significantly associated with parents; perceptions, as shown in Table 3.

**Table 3:** Associations between PPLLIS and different socio-demographic characteristics.

**Variable**	**Group 1**	**Group 2**	**Group 3**	***P*-value**
	***N* = 32**	***N* = 33**	***N* = 35**	**(Pearson chi-square)**
**Parent's sex**			
Male	7 (38.8%)	9 (50%)	2 (11.1%)	0.058
Female	25 (30.5%)	24 (29.3%)	33 (40.2%)	
**Province**			
Aseer	1 (50%)	1 (50%)	0 (0%)	0.777^*^
Eastern	25 (32.5%)	27 (35%)	25 (32.5%)	
Qassim	1 (25%)	2 (50%)	1 (25%)	
Makkah	2 (25%)	2 (25%)	4 (50%)	
Madinah	2 (40%)	1 (20%)	2 (40%)	
Riyadh	1 (25%)	0 (0%)	3 (75%)	
**Highest education of the parent**			
None	1 (100%)	0 (0%)	0 (0%)	0.148^*^
High school	6 (60%)	3 (30%)	1 (10%)	
University and higher	25 (28.1%)	30 (33.7%)	34 (38.2%)	
Older sibling age (Mean ± SD)	13.53 ± 8.46	12.87 ± 7.08	10.57 ± 7.64	0.263
**Older sibling sex**			
Male	16 (29%)	16 (29%)	23 (42%)	0.256
Female	16 (35.6%)	17 (37.8%)	12 (26.7%)	
Younger sibling age (Mean ± SD)	4.92 ± 5.17	4.59 ± 3.03	3.93 ± 3.08	0.566
**Younger sibling sex**			
Male	15 (27.2%)	18 (32.7%)	22 (40%)	0.354
Female	17 (37.8%)	15 (33.3%)	13 (28.9%)	
**Languages spoken at home**			
Arabic	23 (35.4%)	22 (33.8%)	20 (30.8%)	0.343
Arabic + other	9 (25.7%)	11 (31.4%)	15 (42.8%)	
**Languages spoken by the child**			
Arabic	23 (38.3%)	19 (31.6%)	18 (30%)	0.260
Arabic + other	9 (22.5%)	14 (35%)	17 (42.5%)	

* *Fisher's exact test*.

### Parents' Behaviors

In the questionnaire, parents were asked about the most important things to help the child learn to read and write. We categorized the answers into five categories, each containing different responses, as shown in Table 4. There was a significant relationship between parents' total score on the PPLLIS and parents' participation in literacy activities (*N* = 62, *P* = 0.002). This suggests that those participating in literacy activities with their kids were more holistic parents, scoring in the top one-third on the PPLLIS, as shown in Table 5.

**Table 4:** The Parents' Behaviors of Literacy Learning Interview Schedule (PPLLIS) score.

**Category**	***N***	**%**
**1. Direct teaching activities (total responses** **=** **46)**		
- Teach the alphabet (help children recognize letters and sounds and to Write the alphabet)	13	28.3
- Help child write his/her name and the name of things (animals,...)	12	26.1
- Use workbooks with their child	14	30.4
- Teach him the right way to hold a pen and reading by using his finger	7	15.2
**2. Participation in literacy activities (total responses** **=** **62)**		
- Read to them (mostly stories)	18	29.0
-Play games with them (tracing using bullets, coloring the alphabets,…)	27	43.5
-Write with them (alphabet, grocery lists, and notes)	17	27.4
**3. Encouragement/demonstrating/valuing of literacy (total responses** **=** **37)**		
- Provide books, workbooks, and stories for the child	10	27.0
-Provide literacy computer games with stories and letters and cassette tapes	8	21.6
- Let children see parents reading and writing	7	18.9
- Encouraging by words or giving gifts	12	32.4
**4. Knowledge development (total responses** **=** **31)**		
- Talk to them/answer their questions and discussing different things	10	32.3
-Draw pictures with them	16	51.6
-Go on outings with them especially to libraries	5	16.1
**5. Other (total responses** **=** **12)**		
- Reading the sign and advertisement boards when going out	4	33.3
-Start at young age	5	41.7
- Support continuously with patience	2	16.7
-Make new songs on spot	1	8.3

**Table 5:** Partial correlations between parents' behaviors and parents' total score on the PPLLIS.

**Parents' behaviors**	**Parents beliefs**			
	***N***	**df**	***r***	***P*-value**
Direct teaching activities	46	43	−0.149	0.063
Participation in literacy activities	62	59	0.314	0.002^*^
Encouragement/demonstrating/valuing of literacy	37	34	−0.191	0.057
Knowledge development	31	28	0.004	0.972
Other	12	9	0.013	0.928

* *Statistically significant*.

**Figure 1:**
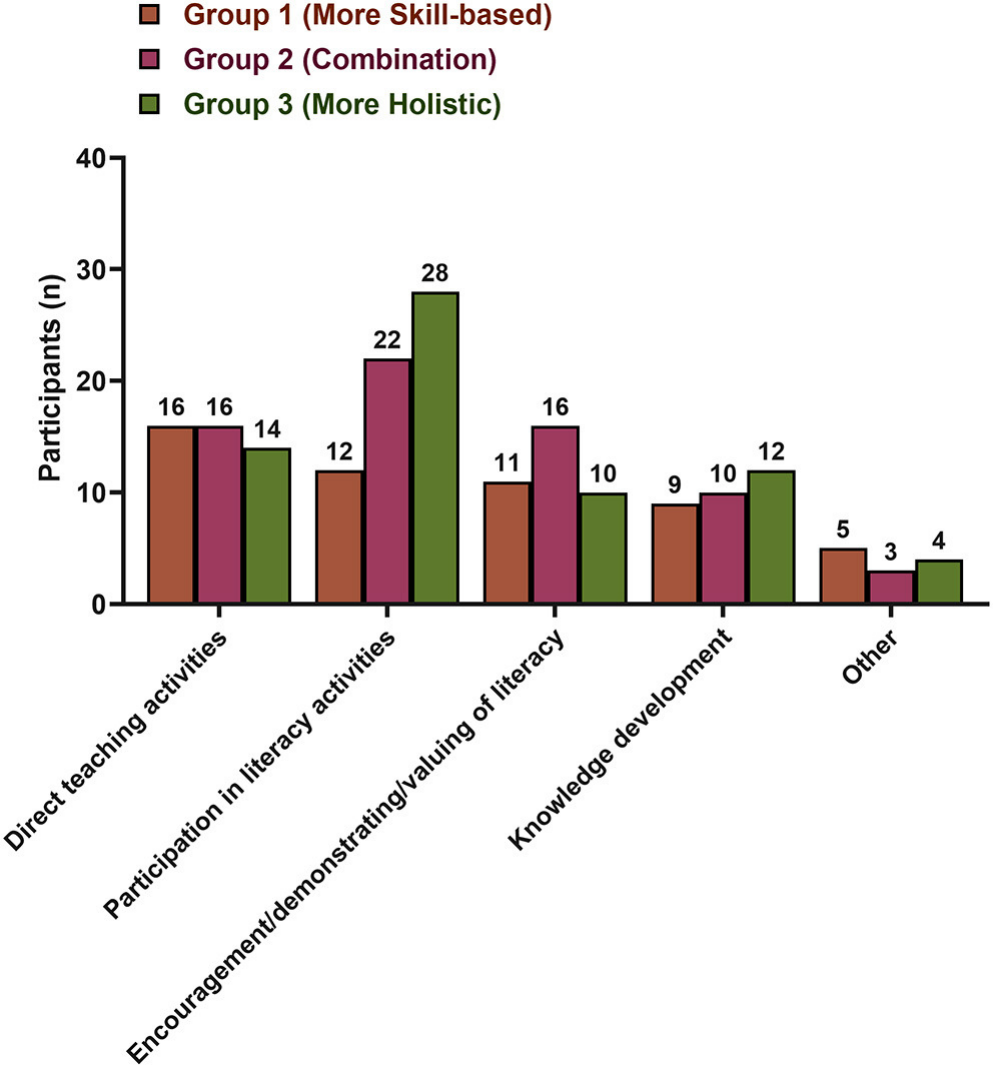
Parents' behaviors and the grouping of parents' beliefs.

The division of parents' beliefs into the three groups showed that parents with more skills-based beliefs were less likely to participate in activities grouped in the “participation in literacy activities” category, such as reading to children, playing games with them, and writing with them (Figure 1). Nearly twice as many parents in the groups 2 and 3 reported these activities types compared to the group 1. Within our sample, no clear trends could be identified.

## Discussion

Our aim in this investigation is to assess the behaviors and beliefs of parents about their children's literacy development in Saudi Arabia. Our results showed that the estimated PPLLIS scores for the included participants were higher, indicating overall holistic beliefs than the overall scores of skills focused on perception. We also found that the place of residency (province) and the high educational status of the parents were the only two significant factors associated with having high PPLLIS scores.

Our results are consistent with the previous investigation by Lynch et al. (16) that showed that higher educational levels were also associated with firmer holistic beliefs. Earlier research by Fitzgerald et al. (17) also reported that having more skill-based beliefs were significantly related to well-educated parents. On the other hand, Stipek et al. (18) reported that parents with higher educational levels were significantly associated with less support for didactic methods, including flashcards. In comparison, the opposite was noticed with parents with lower educational levels. Moreover, according to a previous report by the Organization for Economic Cooperation and Development, the parenteral different socioeconomic factors were not as significant as reading enjoyment in achieving higher educational success rates among children (19). The most reported literacy behaviors were reading stories, talking/discussing with them, and using picture clues grouped in the “Participation in literacy activities” category. In the same context, the current evidence supports graph phonemic (phonics, sounding out words, books with structured vocabulary, and spelling) and constructivist (readers rely on their general knowledge, the language, pictures, and the context of the text) as primary dimensions of beliefs about how children should be taught to read (20).

Our results also showed that participants with the highest scores believed that being more involved with their children in reading, writing, and making shopping lists can enhance literacy according to the PPLLIS score. Senechal and LeFevre previously reported that reading was the most sensitive subject that can be affected by the involvement of parents with their children (21). Furthermore, it has been reported that successful reading practices can, in turn, enhance the educational outcomes of other school subjects (22). Previous investigations have demonstrated that daily activities are significantly associated with many benefits on children in the home, such as language comprehension, reading achievement, more frequent expression of the acquired linguistic skills, and enhanced attitude about reading in schools (23, 24). Besides, it has also been previously reported that being involved with the child's literacy-relevant practices was a more significant predictor for educational success than parenteral family size, social class, and level of education (25). However, we did not find a significant correlation between direct teaching activities or valuing or encouraging literacy practices. On the other hand, Feinstein and Symons reported that achievement and success at 16 years of age were significantly associated with parents' enthusiasm and interest in their children's education and related outcomes (26).

Previous investigations have also reported that the early involvement of parents in literacy practices with their children can significantly enhance the educational outcomes and is usually associated with more lasting effects (27). However, it should be noted that although the early inauguration of parents' involvement with children is essential, it is also necessary that participation in the successful habits should also continue in the latter years, even during the adult and teenage years (27). In addition to the educational outcomes, evidence also shows that parents' involvement with children can also positively impact their emotional status, including more enhanced mental health and self-control, in addition to reduced delinquent behaviors (27, 28). Furthermore, the effect of dynamic interaction between the parents and their children cannot be ignored. Previous investigations reported that mother book reading had a positive association with children's development of early literacy skills (16).

We believe that our findings would imply future national investigations and campaigns that should enhance educational outcomes among both the parents and children. Such campaigns should raise awareness about reading for pleasures practices associated with favorable results on all the education parameters, especially for children in need. Moreover, it has been previously reported that children to parents who value reading are more frequently associated with higher motivations for reading for pleasure and enhanced educational outcomes (26). In the same context, a previous investigation by Baker and Scher also reported that parents who read to entertain their children were more likely to have children who enjoy, value reading, and feel sufficient in their reading activities (29).

It is worth reporting that our findings might be limited. First, the study sample and selection of participants might not adequately represent the general population in Saudi Arabia. Therefore, further studies with larger sample sizes and a random broad selection of the participants are encouraged. Moreover, the lack of temporality associated with the cross-sectional design and the potential social desirability bias is another limitation to this study that future investigations should consider. In addition, the majority of the respondents were mothers which do not reflect the actual population of fathers in the whole population.

## Conclusion

Our findings indicate the overall holistic beliefs than the overall scores of skills that focused on the perception among our Saidu population. Parents' beliefs can influence their behaviors toward their children and higher educational levels can significantly influence such outcomes. We also found that parents' participation in literacy activities with their children was correlated considerably with good behavioral scores. For further validation of our evidence, future national studies are encouraged, together with campaigns aiming to increase the degree of awareness among parents about the benefits of reading for joy in enhancing their children's literacy outcomes.

## Data Availability Statement

The original contributions presented in the study are included in the article/Supplementary Material, further inquiries can be directed to the corresponding author.

## Ethics Statement

Written informed consent was obtained from the individual(s) for the publication of any potentially identifiable images or data included in this article.

## Author Contributions

FA contributed to conceptualizing, drafting, data collecting, writing, editing, and rechecking references accordingly.

## Conflict of Interest

The author declares that the research was conducted in the absence of any commercial or financial relationships that could be construed as a potential conflict of interest.

## Publisher's Note

All claims expressed in this article are solely those of the authors and do not necessarily represent those of their affiliated organizations, or those of the publisher, the editors and the reviewers. Any product that may be evaluated in this article, or claim that may be made by its manufacturer, is not guaranteed or endorsed by the publisher.
