# Supplementary material for: Parents' Beliefs and Behaviors About Their Children's Literacy Development: A Cross-Sectional Study in Saudi Arabia
Source: Front Pediatr. 2022 Apr 7;10:708217. doi: 10.3389/fped.2022.708217 (PMC9022964; doi:10.3389/fped.2022.708217)
Supplement: Supplementary file 1 [file Data_Sheet_1.pdf]

EVALUATION

Please summarize the main findings of the study.

Please highlight the limitations and strengths.

Limitations:1. The paper needs very extensive language editing.
2. There were 83 mothers and 17 fathers involved in the study and most of them were from the eastern province of Saudi Arabia (78%). 78% of the participants are from the eastern province, so this may not provide an accurate generalizable result.
3. Their education level ranged from high school completion to doctorate degrees with 90% having a university degree and higher education. This as well is a major limitation to the study.
Please comment on the methods, results and data interpretation. If there are any objective errors, or if the conclusions are not supported, you should detail your concerns.
Limitations:1. The paper needs very extensive language editing.
2. There were 83 mothers and 17 fathers involved in the study and most of them were from the eastern province of Saudi Arabia (78%). 78% of the participants are from the eastern province, so this may not provide an accurate generalizable result.
3. Their education level ranged from high school completion to doctorate degrees with 90% having a university degree and higher education. This as well is a major limitation to the study.
In the results section: Table 2: First 2 rows add up to 99 participants and not to 100.
No information was provided on how the sample size was collected
More information should be provided regarding data collection, i.e centers data collected at, who collected the data, how was it collected.
Check List
Is the English language of sufficient quality?
No
Is the quality of the figures and tables satisfactory?
Does the reference list cover the relevant literature adequately and in an unbiased manner?
Are the statistical methods valid and correctly applied? (e.g. sample size, choice of test)
Yes
Are the methods sufficiently documented to allow replication studies?
No
Are the data underlying the study available in either the article, supplement, or deposited in a repository? (Sequence/expression data, protein/molecule characterizations, annotations, and taxonomy data are required to be deposited in public repositories prior to publication)
Not Applicable
Does the study adhere to ethical standards including ethics committee approval and consent procedure?
Yes
Have standard biosecurity and institutional safety procedures been adhered to?
Not Applicable

Please provide your detailed review report to the editor and authors (including any comments on the Q4 Check List):
Limitations:1. The paper needs very extensive language editing.
2. There were 83 mothers and 17 fathers involved in the study and most of them
were from the eastern province of Saudi Arabia (78%). 78% of the participants are from the eastern province, so this may not provide an accurate generalizable result.
3. Their education level ranged from high
school completion to doctorate degrees with 90% having a university degree and higher education. This as well is a major limitation to the study.
In the results section: Table 2: First 2 rows add up to 99 participants and not to 100.
No information was provided on how the sample size was collected
More information should be provided regarding data collection, i.e centers data collected at, who collected the data, how was it collected.
I don't see any significant results that will add to the existing body of literature, and the way findings are reported don't give any essential findings.
